# Preparation and *In Vitro/In Vivo* Characterization of Porous Sublingual Tablets Containing Ternary Kneaded Solid System of Vinpocetine with β-Cyclodextrin and Hydroxy Acid

**DOI:** 10.3797/scipharm.0912-04

**Published:** 2010-05-17

**Authors:** Mona H. Aburahma, Hanan M. El-Laithy, Yassin El-Said Hamza

**Affiliations:** Department of Pharmaceutics and Industrial Pharmacy, Faculty of Pharmacy, Cairo University, Cairo, Egypt

**Keywords:** Hydroxy acids, Sublimable materials, Sublingual Porous tablets, Vinpocetine, β-Cyclodextrin

## Abstract

The demand for sublingual tablets has been growing during the previous decades especially for drugs with extensive hepatic first-pass metabolism. Vinpocetine, a widely used neurotropic agent, has low oral bioavailability due to its poor aqueous solubility and marked first-pass metabolism. Accordingly, the aim of this work was to develop tablets for the sublingual delivery of vinpocetine. Initially, the feasibility of improving vinpocetine’s poor aqueous solubility by preparing kneaded solid systems of the drug with β-Cyclodextrin and hydroxy acids (citric acid and tartaric acid) was assessed. The solid system with improved solubility and dissolution properties was incorporated into porous tablets that rapidly disintegrate permitting fast release of vinpocetine into the sublingual cavity. The pores were induced into these tablets by directly compressing the tablets’ excipients with a sublimable material, either camphor or menthol, which was eventually sublimated leaving pores. The obtained results demonstrated that the tablets prepared using camphor attained sufficient mechanical strength for practical use together with rapid disintegration and dissolution. *In vivo* absorption study performed in rabbits indicated that the sublingual administration of the proposed porous tablets containing vinpocetine solid system with β-Cyclodextrin and tartaric acid could be useful for therapeutic application.

## Introduction

Vinpocetine, a poorly-water soluble base type drug, is widely used for treatment of disorders arising from cerebrovascular and cerebral degenerative diseases [1]. It is reported to increase the cerebral blood flow to ischemic areas in patients with cerebrovascular disease, to decrease platelet aggregability in patients with transient ischemic attack, and to increase red cells deformability in stroke patients [1]. Although vinpocetine shows considerable therapeutic effects, its usefulness is limited by its very poor bioavailability (∼7%) [2] owing to its poor aqueous solubility [3] and manifested hepatic first-pass metabolism [4]. Consequently, it is highly desirable to incorporate vinpocetine in a formulation that overcome both difficulties leading to higher degree of bioavailability.

Systemic drug delivery through sublingual mucosa is one of the best known methods to bypass hepatic first-pass metabolism as drugs are not exposed to the metabolic enzymes of the liver [5]. On contact with the sublingual mucosa, the drug permeates across the mucosal tissue to reach the systemic circulation directly. An important factor that precedes permeation of drug is its solubilization in aqueous saliva [6]. Vinpocetine may be considered as a suitable candidate for sublingual delivery due to its small dose (5 mg), appropriate partition coefficient (log K_o/w_ = 3.56), and moderate molecular weight (350.46) [7]. However, its sublingual delivery may be extremely challenging due to its poor aqueous solubility.

Cyclodextrins are a group of cyclic oligosaccharides that have been successfully employed in sublingual formulations to improve the solubility and bioavailability of various poorly soluble drugs [8, 9]. For a series of reasons; price, availability, good safety profile, and ability to mask drugs bitter taste [10]; β-Cyclodextrin (βCD) was selected in this study. Moreover, several studies have demonstrated the effectiveness of incorporating hydroxy acids with βCD in order to improve the aqueous solubility of poorly-water soluble basic drugs [11, 12]. Therefore, it seemed of great interest to investigate the effect of adding either citric acid or tartaric acid with βCD on the solubility and dissolution of vinpocetine. These two hydroxy acids were chosen due to their sour palatable taste upon dissolution in the sublingual cavity.

Nevertheless, sublingual delivery systems are compromised by the possibility of the patient swallowing the active substance before it has been released and absorbed locally into the systemic circulation [13]. Hence, this paper introduces a porous tablet system that assures rapid disintegration and release of vinpocetine after its sublingual administration. Porous structure was induced into these tablets by sublimation of either menthol or camphor which was directly compressed with the tablets excipients [14–16].

To the best of our knowledge, up to date, no report has ever dealt with the sublingual delivery of vinpocetine, althought this is highly beneficial from theraputic view point. Accordingly, the objective of this study was to prepare and characterize porous sublingual tablets of vinpocetine. These tablets contained ternary kneaded solid system of vinpocetine with βCD and hydroxy acid that is characterized by maximum solubility and dissolution. Pores that assures rapid disintegration of the tablets in the sublingual cavity were induced into the proposed tablets using sublimable materials. The pharmaco-technical properties, namely weight variation, hardness, friability, content uniformity, wetting time, *in vitro* and oral disintegration time, and *in vitro* dissolution were evaluated for the prepared tablets. Finally, the relative bioavailability of the optimal porous tablets formulation was compared to the commercially available Cavinton^®^ tablets in fasted rabbits using cross over design.

## Results and Discussion

### In vitro dissolution studies

**Fig. 1.**
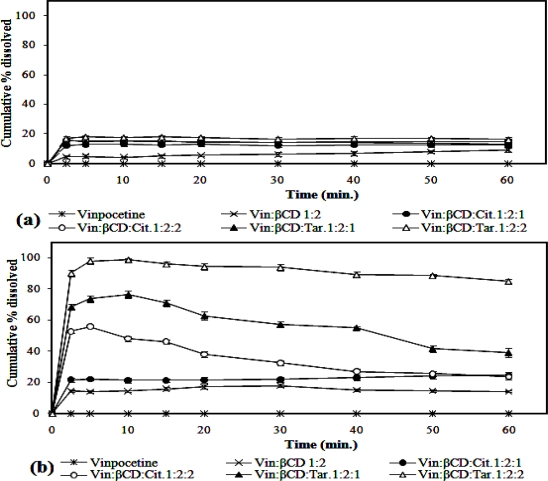
*In vitro* dissolution profiles of vinpocetine (n=3) from its binary and ternary solid systems with β-cyclodextrin and hydroxy acids performed in phosphate buffer of pH 6.8 at 37±0.5°C. (a) Physical mixtures (b) Kneaded solid systems.(Vin: Vinpocetine; Cit: Citric acid; Tar: Tartaric acid).

The *in vitro* dissolution profiles of vinpocetine from the investigated physical mixtures and kneaded solid systems are illustrated in Figure 1.

It is clear that binary and ternary physical mixtures exhibited higher extents of vinpocetine dissolution compared to that of pure vinpocetine, which showed negligible dissolution. This finding agrees well with that mentioned by Ribeiro et al. [17] who attributed this improvement in drug dissolution to the solubilization action of βCD and hydroxy acids operating locally in the aqueous hydrodynamic layer surrounding the drug particles.

Moreover, the kneaded solid systems showed better extents of vinpocetine dissolution compared to their respective physical mixtures probably due to the increase in drug-carrier contact surface as a consequence of the more drastic mechanical treatment imposed during the kneading process as reported in literature [17–20]. Furthermore, the extent of vinpocetine dissolution from its ternary kneaded solid systems was higher than those of the binary kneaded solid systems. This observation may be related to the existence of hydroxy acids which probably created an acidic microenvironment pH more favourable for drug dissolution. Also, it is reported that the hydroxy acids act as a bridge between the drug molecules and βCD molecules by interacting with the basic functional groups of the drug and hydroxyl groups of βCD [21].

It is worth noting that the type of hydroxy acid used had a noticeable effect on vinpocetine dissolution profiles from ternary kneaded solid systems where the latter, prepared with tartaric acid, showed higher extents of drug dissolution than those prepared with citric acid. This might be reasonably attributed to the suitable conformation and the better steric fit that tartaric acid provides for vinpocetine with βCD which consequently led to an increase in the solid system solubility and dissolution. Similar outcome was reported previously in literature [22].

### Solubility studies

Table 1 compiles the solubility, relative increment, and dissolution efficiency data of the prepared binary and ternary physical mixtures and kneaded solid systems. As observed, the aqueous solubility of vinpocetine (0.008 mg/ml at 25°C) increased in all of the prepared binary and ternary physical mixtures and kneaded solid systems. The latter was clearly more soluble than their corresponding physical mixtures and this was related to the increase in the interaction between the drug and βCD as a consequence of the more drastic mechanical treatment during kneading process compared to physical mixing [19].

Moreover, kneaded ternary solid systems were clearly more effective in enhancing vinpocetine solubility than the kneaded binary solid systems. This is probably attributed to the presence of hydroxy acids that gave a synergistic mutual solubility enhancing effect to both vinpocetine and βCD. It is reported that the hydroxyl groups present in hydroxy acids interact with βCD through hydrogen bonds formation and/or modify the hydrogen bonds network of the surrounding water molecules leading to increase in βCD solubility [11].

### Preparation of porous tablets containing vinpocetine

The following additives were selected to prepare vinpocetine porous tablets. Mannitol was employed as a diluent as it has negative heat of solution which imparts a unique cooling sensation and pleasant taste when used in formulations for tablets intended to dissolve in the oral cavity [23]. Ac-Di-Sol was used as a super disintegrant as it has fibrous particles that acts as channels to absorb water allowing rapid disintegration [24]. Camphor and menthol were chosen as sublimable materials, to induce pores into the compressed tablets, as they have the ability to sublimate (i.e., transit from solid phase directly to vapor phase without passing through an intermediate liquid phase). Kollidon CL was included in the formulations as a bioadhesive component in order to increase the contact time of vinpocetine at the sublingual mucosa, thereby reduce the potential intra/inter-individual variability resulting from swallowing the drug [13, 25]. Nevertheless, it is reported that Kollidon CL does not interfere with the rapid disintegration of the tablets [26]. Magnesium stearate was selected as a lubricant owing to its superior lubrication properties. However, its highly hydrophobic nature might build a barrier against rapid disintegration and dissolution of tablets. Accordingly, only 0.25% of magnesium stearate was used as a lubricant for attaining good lubrication without interfering with rapid drug disintegration and dissolution.

### Morphological examination of the prepared porous tablets

**Fig. 2.**
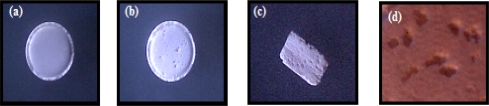
Photographs of (a) Directly compressed vinpocetine tablet containing 10% of camphor before sublimation. (b) Top view, (c) Cross section, and (d) Magnified surface view after removal from vacuum oven.

**Fig. 3.**
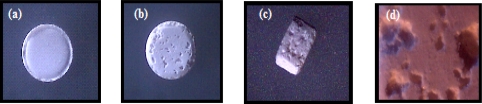
Photographs of (a) Directly compressed vinpocetine tablet containing 20% of camphor before sublimation. (b) Top view, (c) Cross section, and (d) Magnified surface view after removal from vacuum oven.

**Fig. 4.**
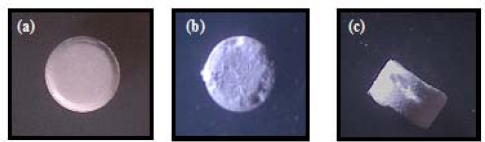
Photographs of (a) Directly compressed vinpocetine tablet containing 10% of menthol before sublimation. (b) Top view, and (c) Side view after removal from vacuum oven.

The morphology of the tablets was examined using an optical microscope and selected photographs are illustrated in figures 2, 3, and 4. It is evident that the sublimation of either menthol or camphor caused a remarkable change in the outer surface of the tablets manifested as pores and cavities. The latter increased in count by the increase in camphor content from 10% to 20% as shown in figures 2b and 3b. Furthermore, those tablets prepared using 10% of menthol showed surface fines and cracks on the tablets surface after sublimation of menthol as depicted in figures 4b and 4c.

### Characterization of the prepared porous tablets containing vinpocetine

Table 2 represents a compilation of the average weight, hardness, and friability of the prepared tablets containing vinpocetine. It was observed that a decrease in the weight of tablets occurred after their removal from vacuum oven. This decrease corresponded to the weight of sublimable materials added which indicates that all of the sublimable material, either camphor or menthol, has been sublimated from the compressed tablets [14].

It was noticed that, all of the prepared tablets of different formulations showed acceptable physical performance regarding tablets’ intactness except those containing 20% of menthol, namely F5 and F10, which were crushed during their removal from vacuum oven and hence were excluded from further study. The hardness of the tablets was decreased after sublimation of the sublimable materials present in them. This was credited to the increase in tablets porosity which consequently decreased tablets’ hardness. The friability values of tablets belonging to formulations F1, F2, F3, F6, F7 and F8 complied with the limits set by the British Pharmacopoeia [27]. Tablets prepared using 20% of camphor, namely F3 and F8, exhibited higher percentage of friability than their counterparts prepared using 10% of camphor, namely F2 and F7. This may be attributed to the presence of more pores in the first than the second. This finding is in agreement with that mentioned by Bi et al. [28] that porosity contributes negatively on tablets’ friability. On the other hand, tablets belonging to formulations F4 and F9 prepared using 10% of menthol were very friable and didn’t comply with the pharmacopoeial limits [27] after sublimation of menthol. This observation might be attributed to the minute particles of menthol employed in these tablets which left numerous small pores after sublimation [14]. Hence, these tablets formulations were excluded from further studies.

Table 3 compiles the average vinpocetine content, wetting time, in vitro and oral disintegration time of selected sublingual tablets. It is obvious that the drug content of all the investigated tablets complied with the pharmacopoeial specifications. It was found that tablets belonging to formulation F3 prepared using 20% of camphor exhibited shorter wetting time and disintegration time (in vitro and oral) than F2 prepared using 10% of camphor. This could be attributed to the greater number of pores formed in these compressed tablets which resulted from the sublimation of the higher percentage of camphor.

Moreover, it was evident that tablets containing Ac-Di-Sol together with pores that were formed after sublimation of camphor, namely F7 and F8, possessed the least wetting time and disintegration time. This might be attributed to the synergistic effect of Ac-Di-Sol as a super disintegrant with the pores caused by camphor sublimation. All of the tested tablets belonging to different formulations were subjected to in vitro dissolution testing except F1 as it showed the longest disintegration and wetting time.

### In vitro dissolution of vinpocetine sublingual tablets

The saliva ordinary maintains the pH of the mouth between 5.6 and 7.8 [13]. Furthermore, literatures performed dissolution studies on sublingual tablets in phosphate buffer of pH 6.8 [8, 9]. Therefore, phosphate buffer of pH 6.8 was indicated as a dissolution medium in dissolution studies.

**Fig. 5.**
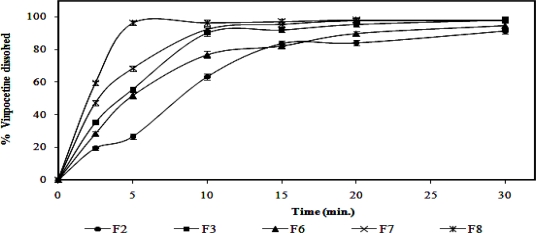
Dissolution profile of vinpocetine (n = 3) from the prepared porous sublingual tablets performed in phosphate buffer of pH 6.8 at 37±0.5°C.

Figure 5 illustrates the dissolution profile of vinpocetine from the prepared porous sublingual tablets. It is obvious that, porous tablets prepared according to the formulation F8 exhibited the highest extent of drug dissolution compared with the other tested tablets of different formulations. This finding might be attributed to the greater number of pores present in these tablets due to sublimation of 20% of camphor in addition to the existence of 5% of Ac-Di-Sol in them resulting in higher rate of tablets disintegration and dissolution. Therefore, this sublingual tablets formulation was subjected to further investigations implicating an *in vivo* absorption study.

### In vivo evaluation of the porous sublingual tablets

**Fig. 6.**
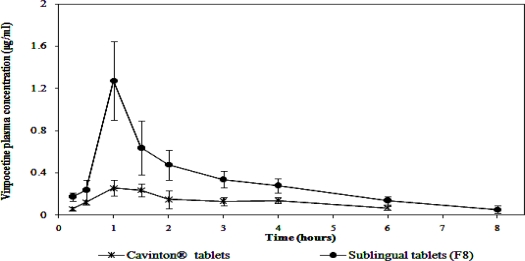
Mean plasma concentrations of vinpocetine (mean ±SD, n = 12) following oral administration of Cavinton^®^ tablets and the sublingual administration of porous tablets belonging to formulation F8.

The plasma profiles of vinpocetine in twelve rabbits following oral administration of Cavinton^®^ tablets and sublingual administration of the sublingual porous tablets belonging to formulation F8 are represented in figure 6 and the corresponding pharmacokinetic parameters are collectively presented in table 4.

Taking Cavinton^®^ tablets as the reference product, the relative bioavailability of vinpocetine after sublingual administration of the selected sublingual tablets, containing vinpocetine kneaded solid system with βCD and tartaric acid in 1:2:2 molar ratio, respectively, was 306.69. This increase in vinpocetine’s bioavailability after sublingual administration is most probably due to the avoidance of its first-pass metabolism. Moreover, it is reported that βCD present in the incorporated kneaded product have the ability to interact with the macromolecules of sublingual membrane causing marked improvement in the drug sublingual absorption [29].

Paired t test was performed in order to statistically analyze the difference between the pharmacokinetic parameters of the investigated sublingual tablets and Cavinton^®^ tablets. It was found that there were significant differences in Cp_max_ and AUC_(0–10)_ between Cavinton^®^ tablets and the tested sublingual tablets. However, a decrease in the T_max_ was observed in case of the sublingual tablets compared to the conventional commercial tablets. This difference was found to be statistically non significant.

## Conclusion

The present study was carried out to develop porous tablets for sublingual administration of vinpocetine. The major obstacle against sublingual delivery of vinpocetine was its low aqueous solubility, which was overcome by kneading the drug with βCD and tartaric acid in a simple and easy-to-scale-up formulation strategy. Porous structure, aiding rapid disintegration and dissolution of tablets in the sublingual cavity, was successfully achieved after camphor sublimation from the directly compressed tablets by means of vacuum oven. Therefore, it is reasonable to say the adopted formulation strategy to prepare porous sublingual tablets could be of great potential for drugs suffering from extensive first-pass metabolism.

## Experimental

### Materials

Vinpocetine was provided by Medical Union Pharmaceuticals Co. (Abu Sultan, Egypt). β-cyclodextrin (MW 1135), camphor, and menthol were purchased from Sigma Chemical Company (St. Louis, USA). Citric acid and tartaric acids were obtained from El-Nasr pharmaceutical Co. (Abu Zaabal, Egypt). Mannitol was donated by Roquette Corp. (Lesterm, France). Kollidon CL (cross-linked polyvinylpyrrolidone) was purchased from BASF corp. (Ludwigshafen, Germany). Ac-Di-Sol (crosscarmellose sodium) was purchase from FMC Corp. (Philadelphia, USA). Magnesium stearate was obtained from Prolabo (Paris, France). Cavinton^®^ tablets (5 mg vinpocetine) were purchased from ACAPI (Badr City, Egypt). HPLC grade methanol and acetonitrile were obtained from Romil Limited (London, United Kingdom). All other reagents used were of analytical grade and were used as received.

### Methods

#### Preparation of vinpocetine-βCD binary and ternary solid systems

With the aim of improving the poor aqueous solubility and dissolution of vinpocetine; binary solid systems of vinpocetine with βCD in 1:2 molar ratios, and ternary solid systems with βCD and hydroxy acids (tartaric or citric acid) in 1:2:1 and 1:2:2 molar ratios were prepared by kneading method. These components in their adequate molar ratios were mixed together followed by kneading them thoroughly with the least amount of water for 20 minutes in a glass mortar. The formed homogenous mixture was left to dry for 48 hours in a desiccator maintained at room temperature. The corresponding physical mixtures were prepared in the same molar ratios as in the prepared kneaded solid systems for comparative purpose.

#### In vitro dissolution testing

Preliminary dissolution tests, aimed at selecting the solid system with superior dissolution properties, were performed according to the dispersed amount method [30]. Binary and ternary physical mixtures and their corresponding kneaded solid systems equivalent to five milligrams of vinpocetine were prepared and were added to 100 ml of phosphate buffer of pH 6.8, thermostated at 37±0.5°C, in a 200 ml beaker. A magnet was immersed in the beaker and rotated at 50 rpm. At appropriate time intervals, samples from the dissolution medium were withdrawn, filtered through Millipore filter of 0.45 μm pore size, and spectrophotometrically assayed at λ_max_ 314.4 nm for drug content. The removed samples were replenished with equal volumes of dissolution medium to keep the dissolution medium volume constant. The dissolution efficiency of vinpocetine from the prepared solid systems, after 60 minutes, was calculated according to Khan [31].

#### Solubility studies

Solubility studies were performed by adding excess of vinpocetine, binary and ternary kneaded solid systems, and their corresponding physical mixtures to 10 ml distilled water in glass vials and were shaken in a thermostatically controlled shaking water bath maintained at 25±0.5°C. After 48 hours, aliquots were withdrawn from vials, filtered through 0.45μm Millipore filter, and spectrophotometrically measured for estimating drug content.

#### Preparation of porous tablets containing vinpocetine

Vinpocetine tablets were prepared by direct compression technique according to the compositions presented in Table 5.

Vinpocetine: βCD: tartaric acid kneaded solid system prepared in 1:2:2 molar ratios, respectively, that showed the highest solubility and extent of drug dissolution was incorporated in the proposed porous sublingual tablets. It was mixed with Kollidon CL for 20 minutes using a pestle in a glass morter. Following that, the calculated amount of mannitol was added to the above mixture and blended together for additional ten minutes. Prior to compression, the sublimable material, either camphor or menthol, was added to the tablets mixture and was mixed with for 20 minutes. Lastly, magnesium stearate was incorporated to the previous mixture and mixed for additional 10 minutes. Tablets of 200 mg were prepared using a single punch tablet press machine equipped with 8 mm punch and die. The tablets were then placed in a vacuum oven adjusted at 60°C for 2 hour to eliminate camphor or menthol by sublimation leaving many pores where they previously existed in the compressed tablets [14]. The formed pores or cavities allow rapid penetration of saliva into these tablets leading to fast disintegration when placed in the oral cavity.

#### Morphological examination of the prepared tablets

The morphological characteristics of the tablets after sublimation of the contained sublimable materials in the vacuum oven were examined using an optical microscope.

#### Physical characterization of the prepared tablets

The mean weight of twenty tablets, randomly selected from each formulation, was determined. Following that, the tablets were placed in a vacuum oven at 60°C for 2 hours to remove camphor or menthol by sublimation and the tablets were reweighed to deduce the weight loss [16]. Moreover, the mean hardness of ten tablets, selected from each formulation, before and after introducing into the vacuum oven, was evaluated and expressed in Kilograms. Added to that, the friability of the tablets, after removal from the vacuum oven, was performed according to British Pharmacopoeia [27]. It should be noted here that tablets which passed the pharmacopoeial demands relevant to weight variation, hardness, and friability tests were subjected to the subsequent tests.

#### Wetting time test

The wetting time of the prepared tablets was measured using a simple procedure. In this method, a filter paper of 10 cm diameter was placed in a petri dish, with diameter of 10 cm, followed by addition of ten milliliters of water containing methylene blue. A tablet from each formulation was carefully placed on the surface of the filter paper. The time required for water to reach the upper surface of the tablets was recorded and taken as the wetting time. To check for reproducibility, these measurements were carried out in triplicates and the mean values were calculated [15].

#### Disintegration time test

The in vitro disintegration time of six tablets from each formulation was determined using USP disintegration tester [27]. However, for measuring the oral disintegration time of the prepared tablets, time needed for a tablet to completely disintegrate in subject’s mouth, three healthy volunteers were asked to rinse their mouths with distilled water. A tablet was placed below the tongue of each volunteer and immediately a stop watch was started. Then, the volunteers were allowed to move the tablets against the lower palate of the mouth with the base of their tongue and to cause a gentle tumbling action on the tablets without biting on them or turning them from one side to other. Immediately, after the last noticeable granules had disintegrated, the stop watch was stopped and the recorded time was taken as the oral disintegration time [32].

#### Content uniformity

The uniformity of vinpocetine content in different tablets was determined by crushing ten tablets from each formulation and determining the content of each tablet individually [27]. The weight of each tablet was dissolved in 100 ml of 0.1N HCl. The solution was then filtered, properly diluted and the absorbance was spectrophotometrically measured at 314.8 nm and then vinpocetine content of each tablet was calculated.

#### In vitro dissolution of vinpocetine sublingual tablets

The dissolution of vinpocetine from its tablets was performed in 200 ml phosphate buffer of pH 6.8, maintained at a temperature of 37±0.5°C, using the USP Dissolution Tester, Apparatus I, at rotation speed of 50 rpm. The dissolution test was done by placing a tablet from each formulation, containing vinpocetine kneaded solid system equivalent to 5mg vinpocetine, in the dissolution basket. Aliquots from the dissolution medium were withdrawn at selected time intervals, filtered through Millipore filter membrane of 0.45 μm pore size, and analyzed for vinpocetine content by measuring their absorbances at 314.4 nm. All the withdrawn samples were replenished with equal volumes of phosphate buffer of pH 6.8 to keep the dissolution volume constant.

#### In vivo evaluation of the prepared porous sublingual tablets

Twelve healthy male albino rabbits (2–2.3 kg) were purchased from the laboratory animal center of faculty of Pharmacy (Cairo University, Egypt). The rabbit was selected as an animal model for absorption studies due to its convenient size, which allows for sublingual administration and blood sample volumes that are sufficient for quantitative analysis. In addition, rabbits have been described as one of the few laboratory animals that do not have keratinized mucosa, thus closely resembling human sublingual mucosal tissue [5, 33].

The rabbits were divided into two groups, each composed of six rabbits. The study was conducted in a cross-over manner with a wash out period of one week. All of the rabbits were healthy during the period of the experiment. The rabbits were fasted overnight but had free access to water. The investigated sublingual porous tablets were administrated using the same procedures adopted by Mannila et al. [8, 9]. Whereby the rabbits were anaesthetized and positioned on a table with the lower jaw supported in a horizontal position. Rabbits’ tongues were carefully lifted with tweezers and the selected sublingual tablet containing vinpocetine complex was placed carefully under the tongue. The rabbits were kept anaesthetized for at least three hours to ensure the maintenance of the tablet in the sublingual cavity without escaping down the GI tract. Cavinton^®^ tablets were also orally administered to the rabbits by tube feeding with the aim of comparing the bioavailability of sublingual tablets to that of oral tablets.

The blood samples (3 ml) were withdrawn via the marginal ear vein into heparinized tubes at specified time intervals and the plasma was separated immediately by centrifugation at 3000 rpm for 10 min followed by storing at −20°C until being assayed for vinpocetine content. The concentration of vinpocetine in rabbit's plasma samples was determined using the HPLC procedures reported by the Abd Elbary et al. [34].

#### Pharmacokinetic data analysis and bioavailability evaluation

The maximal plasma concentration (C_max_) and the time to reach maximum plasma concentration (T_max_) were directly obtained from the plasma data and were compared for statistical significance by Paired t test. The area under the plasma concentration-curve (AUC_0–10_) was also calculated using the trapezoidal rule and the bioavailability of the investigated sublingual tablets to Cavinton^®^ tablets was calculated.

## Figures and Tables

**Tab. 1.** Aqueous solubility (mg/ml ±SD, n=3) at 25°C, relative increment, and dissolution efficiency of vinpocetine from the prepared solid systems.(Vin: Vinpocetine; Tar: Tartaric acid; Cit: Citric acid).

**Composition**	**Physical mixtures**			**Kneading solid systems**		

	**Solubility mg/ml± SD**	**R.I.^a^**	**D.E.^b^**	**Solubility mg/ml± SD**	**R.I.^a^**	**D.E.^b^**
**Vin:βCD (1:2)^c^**	0.02 ± 0.01	2.8	6.18	0.21 ± 0.08	26.7	15.19
**Vin:βCD:Tar (1:2:1)^c^**	2.87 ± 0.1	359.0	13.86	5.64 ± 0.6	705.6	56.51
**Vin:βCD:Cit (1:2:1)^c^**	2.46 ± 0.4	307.6	12.27	3.93 ± 0.3	491.8	22.00
**Vin:βCD:Tar (1:2:2)^c^**	3.45 ± 0.2	431.2	16.71	7.30 ± 0.8	913.2	90.30
**Vin:βCD:Cit (1:2:2)^c^**	2.92 ± 0.3	364.8	14.32	4.09 ± 0.4	512.2	34.42

a Relative Increment (R.I.) = ratio between drug solubility in the prepared solid system to that of drug alone.

b Dissolution Efficiency (D.E.)= calculated from the area under the dissolution curve at 60 minutes.

c The values between brackets represent the molar ratio of vinpocetine: βCD: hydroxy acid.

**Tab. 2.** Average weight, hardness, and friability of the prepared tablets.

**Formulation**	**Average weight^a^ (mg ± SD)**	**Average weight^b^ (mg ± SD)**	**Hardness^a^ (Kg ± SD)**	**Hardness^b^ (Kg ± SD)**	**Friability (%)**
**F1**	200.09 ± 0.09	199.97 ± 0.04	6.96 ± 0.28	7.18 ± 0.17	0.13
**F2**	200.04 ± 1.02	180.07 ± 1.26	7.11 ± 0.13	5.28 ± 0.21	0.32
**F3**	199.94 ± 0.08	161.20 ± 1.27	7.01 ± 0.32	4.13 ± 0.43	0.65
**F4**	198.06 ± 1.12	183.40 ± 2.54	6.87 ± 0.45	1.71 ± 0.38	2.95
**F5**	200.03 ± 1.08	Undetermined*			
**F6**	200.10 ± 1.34	198.70 ± 1.27	6.11 ± 0.15	6.17 ± 0.31	0.16
**F7**	199.94 ± 1.10	181.20 ± 1.08	5.93 ± 0.53	4.23 ± 0.28	0.42
**F8**	200.01 ± 1.07	158.90 ± 0.57	5.89 ± 0.07	3.64 ± 0.21	0.72
**F9**	199.97 ± 1.21	184.81 ± 2.71	5.87 ± 0.42	0.98 ± 0.42	2.73
**F10**	199.95 ± 0.08	Undetermined*			

a Before sublimation;

b After sublimation;

* The tablets were broken after sublimation of 20% of menthol in vacuum oven.

**Tab. 3.** Average drug content, wetting time, *in vitro* disintegration time, and oral disintegration time of the prepared tablets.

**Formulation**	**Average drug content (%±SD)**	**Wetting time (sec.)**	***In vitro* disintegration time (sec.±SD)**	**Oral disintegration time (sec.±SD)**
**F1**	98.05 ± 0.84	> 300	785.30 ± 16.08	447.42 ± 12.47
**F2**	95.49 ± 0.62	95.46	135.20 ± 8.24	63.49 ± 4.15
**F3**	93.82 ± 0.44	68.28	98.74 ± 4.21	54.12 ± 2.87
**F6**	94.53 ± 0.66	65.91	94.61 ± 6.18	47.45 ± 3.18
**F7**	94.07 ± 0.91	53.20	58.18 ± 5.23	38.93 ± 5.72
**F8**	97.18 ± 0.57	40.45	34.51 ± 3.47	23.21 ± 2.24

**Tab. 4.** Summary of pharmacokinetic parameters of vinpocetine following the administration of Cavinton^®^ tablets and sublingual porous tablets of formulation F8.

**Parameter**	**Cavinton^®^ tablets**	**Sublingual porous tablets (F8)**
**Cp_max_ (μg.ml^−1^)^a^**	0.26 ± 0.04	1.34 ± 0.63
**T_max_ (hour)^a^**	1.41 ± 0.80	1.08 ± 0.20
**AUC_(0-t)_ (μg.hr.ml^−1^)^a^**	0.82 ± 0.04	2.53 ± 0.40
**% Relative bioavailability**	–	306.69%

a Mean± Standard deviation.

**Tab. 5.** Composition of vinpocetine sublingual tablets.

**Formulation^a^**	**Vinpocetine Kneaded Solid System (mg)^b^**	**Camphor (%)**	**Menthol (%)**	**Ac-Di-Sol (%)**	**Mannitol up to (mg)**
**F1**	41.66				200
**F2**	41.66	10			200
**F3**	41.66	20			200
**F4**	41.66		10		200
**F5**	41.66		20		200
**F6**	41.66			5	200
**F7**	41.66	10		5	200
**F8**	41.66	20		5	200
**F9**	41.66		10	5	200
**F10**	41.66		20	5	200

a All batches contained 2% Kollidon CL as a bioadhesive agent, and 0.25% magnesium stearate as a lubricant.

b Vinpocetine: βCD: tartaric acid kneaded solid system in 1:2:2 molar ratios equivalent to 5 mg vinpocetine.
